# Associations of Fatty Acids in Cerebrospinal Fluid with Peripheral Glucose Concentrations and Energy Metabolism

**DOI:** 10.1371/journal.pone.0041503

**Published:** 2012-07-24

**Authors:** Reiner Jumpertz, Ana Guijarro, Richard E. Pratley, Clinton C. Mason, Daniele Piomelli, Jonathan Krakoff

**Affiliations:** 1 Klinik für Endokrinologie, Diabetes und Ernährungsmedizin, Charité Universitätsmedizin, Berlin, Germany; 2 Phoenix Epidemiology and Clinical Research Branch, National Institute of Diabetes and Digestive and Kidney Diseases, Phoenix, Arizona, United States of America; 3 Department of Pharmacology, University of California Irvine, Irvine, California, United States of America; 4 Sanford Burnham Medical Research Institute, Orlando, Florida, United States of America; 5 Drug Discovery and Development, Italian Institute of Technology, Genoa, Italy; Montreal Diabetes Research Center, Canada

## Abstract

Rodent experiments have emphasized a role of central fatty acid (FA) species, such as oleic acid, in regulating peripheral glucose and energy metabolism. Thus, we hypothesized that central FAs are related to peripheral glucose regulation and energy expenditure in humans. To test this we measured FA species profiles in cerebrospinal fluid (CSF) and plasma of 32 individuals who stayed in our clinical inpatient unit for 6 days. Body composition was measured by dual energy X-ray absorptiometry and glucose regulation by an oral glucose test (OGTT) followed by measurements of 24 hour (24EE) and sleep energy expenditure (SLEEP) as well as respiratory quotient (RQ) in a respiratory chamber. CSF was obtained via lumbar punctures; FA concentrations were measured by liquid chromatography/mass spectrometry. As expected, FA concentrations were higher in plasma compared to CSF. Individuals with high concentrations of CSF very-long-chain saturated FAs had lower rates of SLEEP. In the plasma moderate associations of these FAs with higher 24EE were observed. Moreover, CSF monounsaturated long-chain FA (palmitoleic and oleic acid) concentrations were associated with lower RQs and lower glucose area under the curve during the OGTT. Thus, FAs in the CSF strongly correlated with peripheral metabolic traits. These physiological parameters were most specific to long-chain monounsaturated (C16∶1, C18∶1) and very-long-chain saturated (C24∶0, C26∶0) FAs. Conclusions: Together with previous animal experiments these initial cross-sectional human data indicate that central FA species are linked to peripheral glucose and energy homeostasis.

## Introduction

Recent advances in lipidomic profiling have shown that specific fatty acid (FA) species in human plasma are associated with adiposity and lifestyle variables, such as smoking, physical activity and diet, while others correlate with hepatic and whole-body insulin sensitivity [1]–[4]. Circulating palmitate (16∶0) is elevated in individuals with coronary heart disease and an increase in dietary intake thereof is associated with lower energy expenditure [5], [6]. It has previously been demonstrated by our group that palmitic acid in the phospholipid fraction of skeletal muscle is associated with increased adiposity in Pima Indians [7]. A number of recent studies have shown that desaturation indexes are also linked to adiposity and insulin resistance. For example, the ratios of palmitic and stearic acid (18∶0) to palmitoleic (16∶1) and oleic acid (18∶1) respectively, have been correlated with adiposity and insulin resistance, while the ratio of dihomogammalinoleic acid (20∶3ω6) to arachidonic acid (20∶4ω6) is negatively associated with obesity and insulin resistance [3], [4], [7]–[11]. Together, these data underscore a possible link of FA saturation in the pathogenesis of the metabolic syndrome. However, FA chain length appears to be an important characteristic which specifies FA regulatory activity. The length of the carbon chain of FAs can be modulated by elongases which show substrate specificity for the degree of saturation [12]. Kitazawa et al. have shown that ELOVL1, 3 and 6 show high substrate specificity for long chain saturated FAs which they can elongate to C22–C26 carbon chain length [13]. Interestingly, a recent study in mice by Zadravec et al. has demonstrated that ablation of elongase 3 (ELOVL3), leading to decreased very-long-chain saturated FAs, protects mice from diet-induced obesity, an effect largely attributable to increased resting metabolic rates [14]. However, whether this was a central or a peripheral effect has not been elucidated.

Recent studies in rodents do indicate a potential role for FA sensing in the central nervous system (CNS), specifically in the hypothalamus, which in turn regulates peripheral glucose and energy homeostasis [15], [16]. These data support the novel concept that lipids may be sensed in the central nervous system leading to profound changes in peripheral metabolism and hunger. Haywood et al. even showed that central but not peripheral lipid infusion augments the counter-regulatory secretion of epinephrine and glucagon in response to hypoglycemia [17]. Moreover, central administration of specific FA species such as oleic acid markedly improves insulin action, inhibits glucose production and reduces food intake in rats [18], [19]. Interestingly, this effect appears to be dependent on chain length and degree of saturation of the FA species being administered. Intervention studies have also provided evidence that inhibition of hypothalamic FA synthesis triggers fatty acid oxidation in skeletal muscle and potently increases whole body energy expenditure underscoring the importance of a brain-muscle axis in energy homeostasis in rodents [20]–[23].

Together, these animal data indicate that FAs in the CNS may be regulators of peripheral glucose and energy metabolism. Therefore, we explored associations of individual FA species in human CSF and plasma with metabolic features in a metabolically phenotyped group of individuals. We hypothesized that very-long-chain saturated FAs would be associated with lower resting metabolic rates and that monounsaturated FAs (such as oleic acid) in the CSF would be associated with lower glucose concentrations during an oral glucose tolerance test. Indeed, the data presented in this manuscript demonstrate that in humans FA species in the CNS stratified by chain length and degree of saturation are associated with peripheral metabolism.

## Materials and Methods

### Study Outline

This is an analysis of paired plasma and cerebrospinal fluid (CSF) samples collected in our research unit during a study investigating the role of leptin in body weight regulation. Thirty-two non-smoking volunteers (Caucasian: 15, American Indian 13, African American: 4), healthy by history, physical examination and standard laboratory tests were admitted to our clinical research unit for 6 days on a weight maintaining diet. On day 2, body composition was measured by dual-energy X-ray absorptiometry and glucose regulation was determined by an oral glucose tolerance test (OGTT). On day 3, volunteers entered a metabolic chamber for measurements of 24-hour energy expenditure (24EE) and sleep energy expenditure (SLEEP). On day 5, lumbar punctures were performed for collection of CSF, and paired plasma samples were drawn. Individuals were discharged on day 6. CSF was available in 29 of the admitted subjects and 26 had data available from the metabolic chamber. All subjects provided written informed consent. The protocol and consent form were approved by the Institutional Review Board of the National Institute of Diabetes and Digestive and Kidney Disease.

### Oral Glucose Tolerance Test

After an overnight fast subjects were given a 75 g oral glucose load. Blood samples were drawn at 0, 30, 60, 120, and 180 min for measurement of plasma glucose and insulin concentrations.

### Whole-room Calorimetry

Energy expenditure (EE) was measured in a respiratory chamber in 26 individuals with CSF samples available, as previously described by our group [24]. Briefly, volunteers entered the chamber at 0745 h after an overnight fast and remained therein for 23 h. Meals were provided at 0700, 1100, and 1600 h, and an evening snack was provided at 1900 h. The rate of EE was measured continuously by the rate of CO_2_ production and O_2_ consumption, calculated for each 15-min interval, and then averaged for the 24-h interval (24EE). SLEEP was defined as the average energy expenditure of all the 15-min periods between 2330 and 0500 h during which spontaneous physical activity (assessed by motion radar) was <1.5%. The respiratory quotient (RQ) was calculated by the mean ratio of CO_2_ production and O_2_ consumption over the 24-hour interval.

### Lumbar Puncture and Plasma Sampling

Lumbar puncture was performed in the morning after a 12-hour overnight fast. Volunteers were placed in a sitting position with maximal flexion of the back. After infiltration with 1% lidocaine, lumbar puncture was performed with sterile technique at either L4–L5 or L5–S1 inter-space with a 22- or 25-gauge needle. Approximately 8 ml of CSF was collected in each case. CSF (2 ml) was analyzed locally for cell count, glucose and protein concentrations to rule out excessive red blood cells (RBC) contamination or other abnormalities. The remaining 6 ml were aliquoted into polypropylene tubes and quick frozen in liquid nitrogen with subsequent storage at −70°C. During the same time of the lumbar puncture blood was drawn from the cubital vein and centrifuged directly thereafter. Plasma was then stored at −70°C.

### Glucose and Insulin Measurements

Plasma glucose concentrations were determined by the glucose oxidase method (Beckman Instruments, Fullerton, CA, USA). Plasma insulin concentrations were measured by Concept 4 radioimmunoassay (Concept 4; ICN, Costa Mesa, CA, USA) and normalized to the modified Herbert-Lau assay using regression equations.

### Lipid Extraction and Analysis of Fatty Acids

FAs were measured in plasma and CSF samples. One ml of ice-cold acetone containing heptadecanoic acid (17∶0) (Nu-Check Prep, Inc., Elysian, MN, USA) as internal standard was added to 1 ml of plasma or CSF. Proteins were precipitated by centrifugation at 1000*xg* for 20 min at 4°C. The supernatant was collected and the excess of acetone was dried under nitrogen stream. Lipids were extracted using 2 volumes of chloroform and 1 volume of methanol and washed with 1 volume of water. The organic phase were collected and dried under nitrogen. The extract was dissolved in 2 ml of chloroform and fractionated onto small glass columns packed with Silica Gel G (60-Å 230–400 Mesh ASTM; Whatman, Clifton, NJ, USA) as described [25]. FAs were eluted with 2 ml of chloroform/methanol (9∶1, vol/vol), dried under nitrogen and reconstituted in 60 µl of methanol. Thereafter, FAs were quantified by liquid chromatography/mass spectrometry (LC/MS) [26]–[28]. FAs are expressed as percentage of total FAs for correlation analyses and both total concentration and relative representation (%) are given in **Table 1**. Saturated fatty acids (SAFA), monounsaturated fatty acids (MUFA) and polyunsaturated fatty acids (PUFA) were calculated by summing the concentrations of individual FAs of each class if detectable divided by the total concentration of FAs. Long-chain FAs are defined by carbon chain length between 12 and 20 C-atoms. Very long chain FAs are defined by carbon chain length greater than 20. Lipids are defined as hydrophobic substances characterized by their long carbon tails.

**Table 1:** Fatty acid concentrations in CSF and plasma.

Fatty Acid	CSF			Plasma*		*R*- value^†^
	(pmol/ml)		(%)	(pmol/ml)	(%)	
**16∶0**	55.51±43.92	30.67±3.76		3793±838	19.54±1.51	0.14
**18∶0**	74.42±29.37	46.28±10.96		1296±170	7.04±2.05	−0.09
**20∶0**	2.77±1.43	1.66±0.52		20.81±9.07	0.12±0.07	0.06
**22∶0**	0.04±0.06	0.02±0.03		18.62±18.83	0.12±0.17	0.13
**24∶0**	4.05±3.15	2.52±1.75		22.53±13.26	0.13±0.10	0.18
**26∶0**	1.83±1.31	1.19±0.78		8.89±6.23	0.06±0.04	0.05
**16∶1**	2.21±4.74	0.76±0.49		1360±729	6.51±1.84	0.15
**18∶1**	23.80±55.08	7.23±6.05		7588±2225	38.22±3.29	−0.07
**20∶1**	1.09±1.93	0.42±0.23		83.02±22.05	0.43±0.08	0.24
**22∶1**	0.88±0.65	0.54±0.36		18.21±5.81	0.10±0.05	0.23
**24∶1**	0.83±0.49	0.51±0.23		25.58±9.42	0.15±0.07	0.35
**18∶2ω6**	14.82±38.37	3.90±4.29		4043±1240	20.39±1.96	0.20
**18∶3ω3**	0.81±1.59	0.30±0.20		313±156	1.59±0.39	**0.58^§^**
**20∶3ω6**	0.83±1.83	0.27±0.21		94.68±34.11	0.49±0.13	−0.01
**20∶3ω9**	0.10±0.14	0.05±0.03		9.83±5.89	0.05±0.02	0.09
**20∶4ω6**	7.05±19.60	1.67±2.30		486±278	2.50±1.56	0.07
**20∶5ω3**	0.36±0.58	0.15±0.06		38.37±34.43	0.19±0.15	0.10
**22∶5ω3**	0.38±0.59	0.18±0.22		95.42±39.53	0.48±0.13	0.19
**22∶5ω6**	0.49±0.46	0.25±0.18		55.96±18.40	0.29±0.10	0.18
**22∶6ω3**	2.45±4.00	1.10±0.67		229±75	1.16±0.40	**0.48^‡^**
**22∶4ω6**	0.64±0.77	0.23±0.14		89.87±34.42	0.45±0.10	0.06

Fatty acid concentrations are depicted as mean ±SD by their total concentration (pmol/ml) and as percentual representation (%) of all measured fatty acids (FA) (n = 28). *p<0.0001 for comparison between plasma and CSF concentrations of FAs derived from Wilcoxon rank tests; ^†^derived from Spearman correlation tests between FA concentrations in the two compartments. ^‡^p = 0.01, ^§^p = 0.001.

### Statistical Analyses

Statistical analyses were performed using SAS Enterprise Guide 9.1 (SAS Institute, Inc., Cary, NC, USA). Spearman correlation tests were used to test associations of FAs with residuals of adiposity variables adjusted for age and sex. Racial differences were evaluated using analysis of variance (ANOVA) and differences of FA concentrations between compartments by Wilcoxon rank test. Due to the relatively small sample size of individuals with measured CSF, direct adjustment for all covariates that vary with energy expenditure (notably: age, sex, fat mass and most importantly fat free mass) would greatly reduce the degrees of freedom and the ability to detect meaningful associations. To overcome this limitation, we pooled the study group with previously measured energy expenditure and substrate oxidation data from 808 non-diabetic individuals who participated in a longitudinal study of diabetes and obesity (EE group, **Table 2**, for design of this study see [29]). We used linear regression models to adjust 24EE for age, sex, fat mass, fat free mass, race, physical activity, fasting and 2-hour glucose. SLEEP was adjusted for the same confounders except physical activity, as was RQ with additional adjustment for energy balance. Adjustments for glucose were made due to slightly elevated glucose concentrations in the EE group. Residuals for the original study group were then extracted and used to perform correlation analyses with FAs, as has been done previously [30]. Although participants received a similar amount of dietary fat (30%), all significant associations were confirmed in multivariate models with additional adjustment for plasma pentadecanoic acid (15∶0) (data not shown) to account for dietary intake of short- and medium chain SAFAs [11]. This essential odd-numbered saturated FA is almost exclusively obtained from dairy fat and ruminant meat products and correlates well with short- and medium-chain SAFA intake [31]. Unadjusted P-values are provided throughout unless otherwise indicated (BON), with tests significant at 0.05 and tests that remain significant after Bonferroni correction for multiple comparisons indicated separately. Student’s T-tests were used to compare group means for **Table 2**.

**Table 2:** Group characteristics.

	Caucasian	American Indian	African American	*P* *	Study Group	EE Group^‡^	*P* ^†^
**N** (male)	15 (9)	13 (7)	4 (1)	–	32 (17)	803 (327)	–
**Age** (years)	30.8±6.5	28.7±6.4	35.0±8.6	0.26	30.5±6.8	29.6±7.5	0.30
**Weight** (kg)	99.8±21.9	87.2±23.2	115.4±30.1	0.10	96.6±24.5	97.0±26.4	0.55
**Waist** (cm)	109.0±17.5	104.9±18.5	115.3±23.1	0.61	108.0±18.3	108.5±19.1	0.54
**BMI** (kg/m^2^)	34.8±8.1	32.3±7.6	37.6±12.0	0.50	34.2±8.3	34.1±8.9	0.64
**PFAT** (%)	32.6±11.2	31.9±8.9	31.2±13.0	0.97	32.1±10.2	31.9±9.0	0.72
**Fasting plasma glucose** (mg/dl)	84.7±9.0 (4.7±0.5)	82.9±10.8 (4.6±0.6)	88.3±5.4 (4.9±0.3)	0.63	84.7±9.0 (4.7±0.5)	90.1±9.0 (5.0±0.5)	**<0.001**
**2 hour plasma glucose** (mg/dl)	115.3±23.4 (6.4±1.3)	124.3±50.5 (6.9±2.8)	109.9±9.0 (6.1±0.5)	0.67	118.9±34.2 (6.6±1.9)	122.5±30.6 (6.8±1.7)	**0.04**
**24EE** (kcal/day)	2247±258	1996±352	2381±279	0.07	2378±321	2404±444	**0.01**
**SLEEP** (kcal/day)	1572±224	1380±248	1792±155	**0.02**	1538±259	1719±327	**<0.01**

* P-values for comparison across racial groups were derived from ANOVA. ^†^P-values for comparison between the study group and the EE group were computed by Student’s t-test. All data are represented as raw unadjusted group mean ±SD. Glucose values in brackets represent glucose concentrations in mmol/l. ^‡^This group is comprised of individuals from an ongoing longitudinal study from our unit with data on energy expenditure. These data were pooled with energy expenditure data from our study group to allow for adequate inclusion of covariates in regression models; residuals were extracted from this larger group for use in correlation analyses as described in the Materials and methods section. Glucose values in parentheses are given in SI units (mmol/l). Energy expenditure data derive from 26 individuals of the study group. BMI: Body mass index, Pfat: percentage of body fat, 24EE: 24 hour energy expenditure, SLEEP: Sleep energy expenditure.

## Results and Discussion

### Group Characteristics

Group characteristics by race are shown in **Table 2**. Racial groups were well matched for age, adiposity and glucose regulation. Compared with the study group, the EE group (larger group used to calculate residuals) had slightly elevated fasting, 2-hour glucose and EE values.

### Fatty Acid Concentrations in Plasma and Cerebrospinal Fluid

Concentrations of specific saturated (SAFA), monounsaturated (MUFA) and polyunsaturated (PUFA) FAs in plasma and CSF are shown in **Table 1**. As expected, all FA species were by far more abundant in plasma vs. CSF. Of note, except for α-linolenic and docosahexaenoic acid (r = 0.58, P = 0.001; r = 0.48, P = 0.01, respectively), none of the measured FA species were correlated between plasma and CSF. The association of docosahexaenoic acid between the two compartments is consistent with a recent report in elderly patients demonstrating a positive correlation between the representation of docosahexaenoic acid in plasma and brain tissue [32]. However, to date supporting data specifically in humans are scarce and limited in their statistical power, which is why these data need to be viewed as preliminary and interpreted with caution. Nevertheless, this association may support the concept that FAs may act as signaling molecules between the CNS and the periphery and implies that specific lipid transport mechanisms exist across the blood brain barrier (BBB). It is believed that FAs enter the CNS by at least two transport mechanisms: diffusion and/or protein-mediated transport. The regulation of these pathways appears to be dependent on chain length and saturation of the substrate which may support the concept that the transfer of FAs across the BBB is highly regulated [33]. To date the exact regulatory mechanism of FA metabolism at the interface of the BBB is not clear. Using deuterium-labeled FAs Edmond has demonstrated that after peripheral injection, linoleic acid is taken up by the brain, however palmitic, stearic and oleic acid are not [34]. The author concludes that the brain is autonomous in producing most lipids. However, others have postulated that FAs enter the brain from the blood [35]. In humans using ^11^C-labeled arachidonic acid it was shown that peripherally injected FAs can be visualized (after only 15 min) in cranial images [36]. Nevertheless, brain fatty acid uptake from the periphery at physiological levels still awaits final proof and therefore the interpretation of our findings is additionally limited. One reason for the lack of associations of FAs between the peripheral and the central compartment in our study could be that by far the largest portion of plasma FAs are bound to plasma proteins, such as albumin (99%), which could have blunted associations between the respective FA species [33]. Another potential issue which could account for this lack of association is that plasma lipoproteins represent an additional source for FA transport across the BBB. It has been proposed that the lipoprotein lipase located on the cerebral microvessel endothelium can liberate additional FAs from lipoproteins making them available for transport into the brain [37]. Thus, whether associations of respective FA species between the periphery and the CNS also reflect FA trafficking between the two compartments needs to be further explored and in this regard our data need to be viewed as preliminary.

### Race

**Figure 1:**
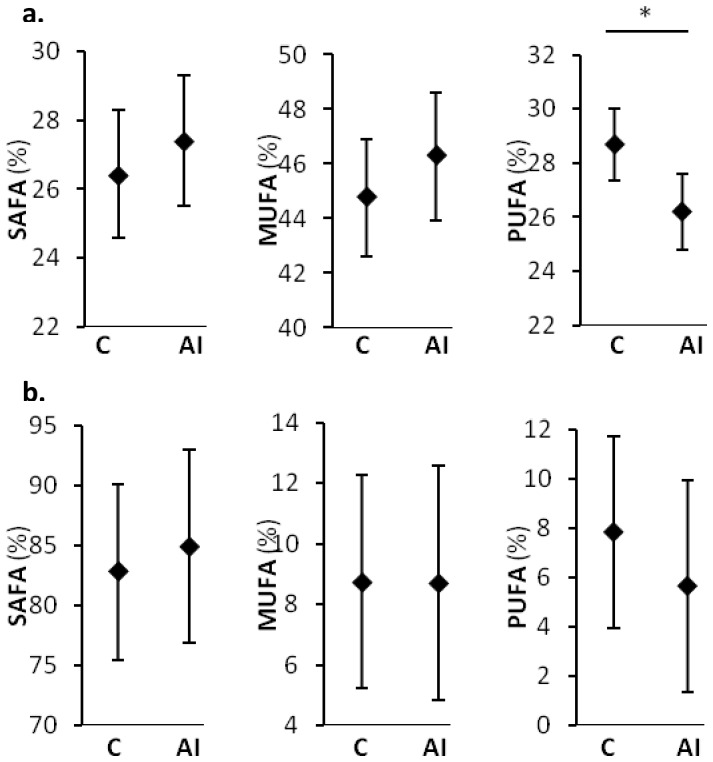
Plasma and CSF fatty acid fractions by race. Percentual abundance of saturated (SAFA), monounsaturated (MUFA) and polyunsaturated (PUFA) fatty acids in plasma (**a.**) and CSF (**b.**) are shown for Caucasian (C) and American Indian (AI) individuals in plasma and CSF. Diamonds (♦) represent least squared means adjusted for age, sex, and body fat and error bars indicate 95% confidence interval. *P = 0.01.

Due to the low number of African-American individuals, race comparisons were made only between Caucasians and American Indians (**Figure 1**). All comparisons were adjusted for age, sex and body fat. In plasma, American Indians had lower PUFA than Caucasians while no racial differences were observed for the SAFA and MUFA fraction. In CSF, no racial differences in any of the FA fractions were observed.

### Adiposity

In plasma the SAFA and MUFA fractions were associated with percent body fat (r = −0.58, P<0.001, r = 0.36, P = 0.04, respectively), while the PUFA fraction was not (r = 0.22, P = 0.22). The association of SAFA with percent body fat remained significant after Bonferroni correction (BON: P = 0.02). However, none of the FA fractions in CSF were associated with adiposity (data not shown).

### Individual Fatty Acid Species and Metabolic Traits

**Figure 2:**
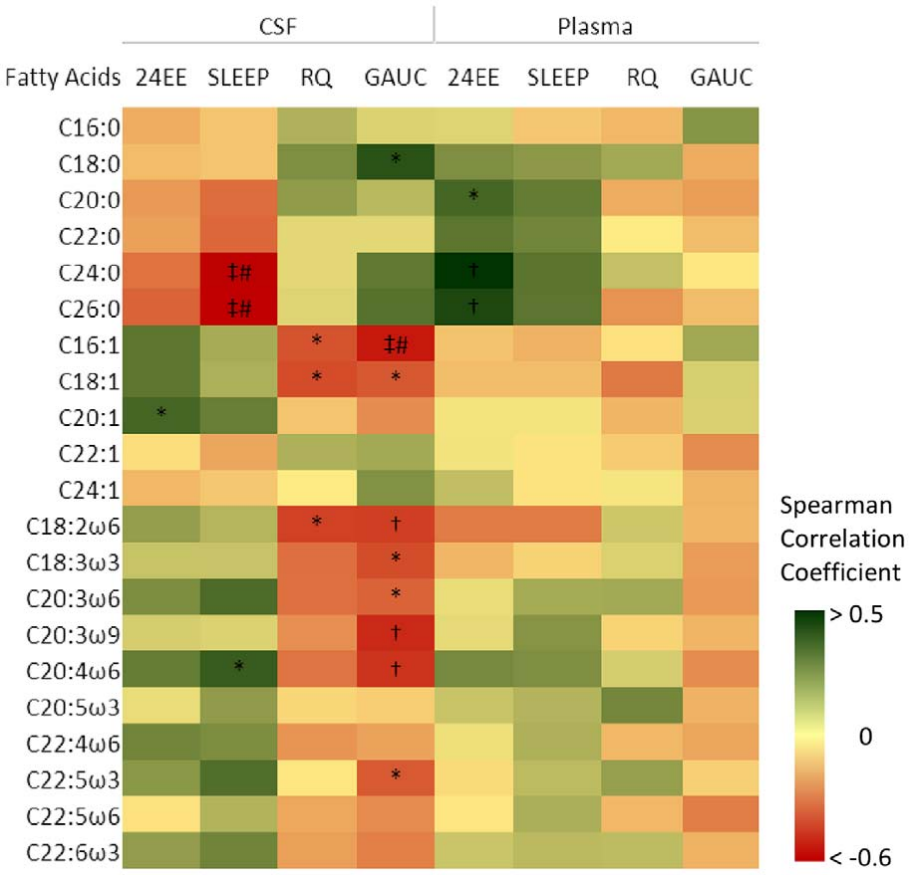
Correlation heatmap of fatty acid species with metabolic traits. Spearman Correlation Coefficients are represented by color codes as illustrated in the side panel. CSF: cerebrospinal fluid; 24EE: 24 hour energy expenditure measured in a metabolic chamber; SLEEP: energy expenditure measured during the sleep phase; RQ: respiratory quotient; GAUC: glucose area under the curve during the oral glucose tolerance test; *p<0.05, †p<0.01, ‡p<0.001, #significant after Bonferroni correction.

**Figure 3:**
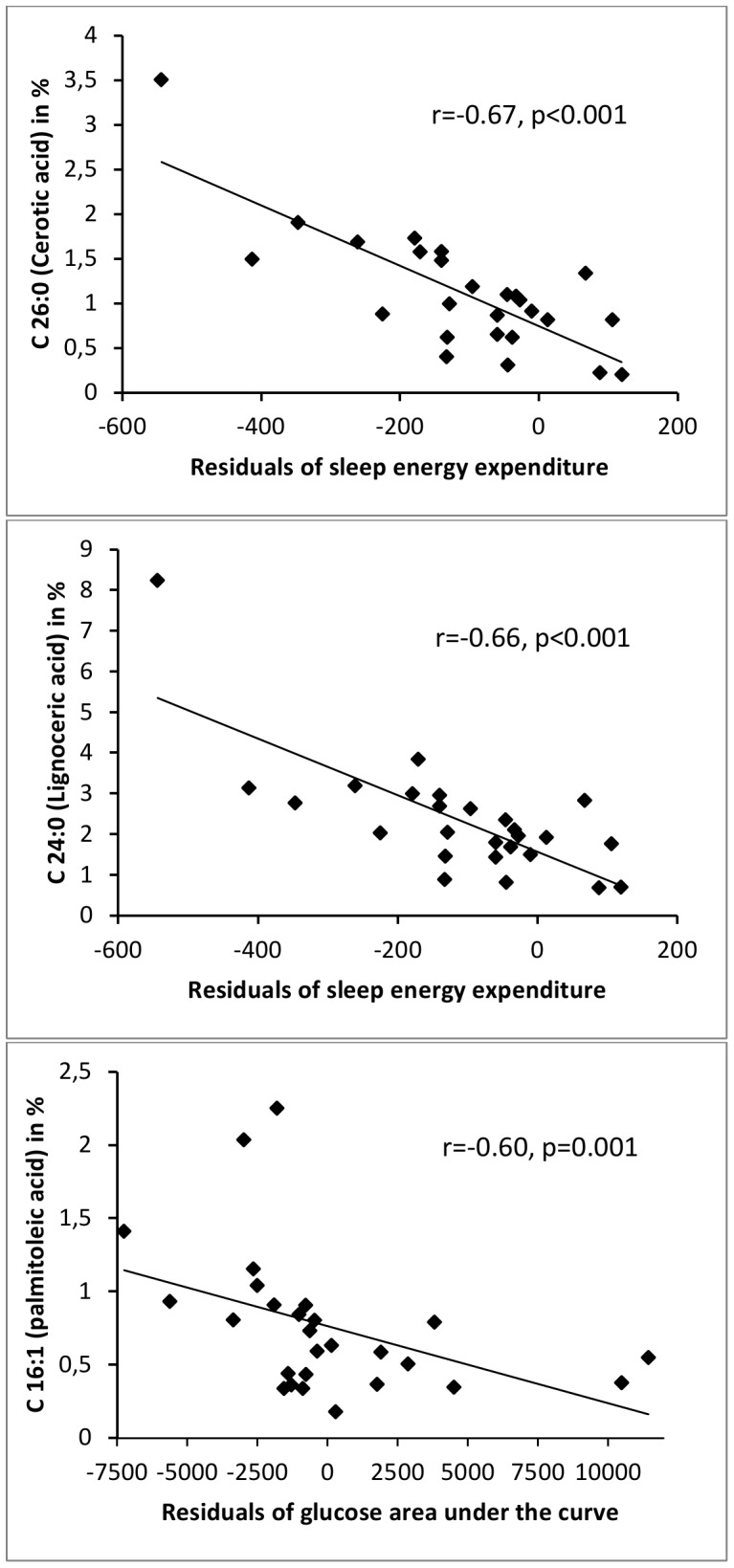
Scatter plots of fatty acids in CSF and metabolic factors. Erucic acid (C26∶0, panel a.) and Lignoceric acid (C24∶0, panel b.) are negatively associated with residuals of energy expenditure during sleep (SLEEP). SLEEP was adjusted for known confounders as described in the Statistical analyses section of the Materials and methods. Panel c. shows the association of palmitoleic acid (C16∶1) with residuals of glucose area under the curve (GAUC). GAUC was adjusted for age, sex and percent body fat. Bonferroni-corrected p-values were a) 0.004, b) 0.004 and c) 0.02.

A correlation heatmap of individual FA species in CSF and plasma with traits of energy expenditure, respiratory quotient (RQ) and glucose metabolism is shown in **Figure 2**. Scatter plots of associations that were significant after Bonferroni correction are shown in **Figure 3**. In plasma, despite moderate positive associations of very-long-chain saturated FAs with 24EE (not significant after Bonferroni correction), the representations of individual FA species were not associated with metabolic traits and no clear correlation clusters with regard to chain length and degree of saturation of the individual FAs were observed. However, in CSF FA species showed strong associations with metabolic traits and this was clustered by chain length and degree of saturation. SAFAs with increased chain length were associated with lower SLEEP (a surrogate for resting metabolic rate) but not with 24EE (**Figure 2** **,** **3a and b**). This observation is consistent with the hypermetabolic phenotype in ELOVL3-ablated mice (which have a lower capacity to produce very-long-chain saturated FAs) specifically during the resting state [**14**]. The hypothalamic melanocortin signaling system is a potential pathway by which very-long-chain SAFAs may affect energy expenditure. Reduced food intake increases the expression of pro-opiomelanocortin (POMC) and decreases cocaine and amphetamine-regulated transcript (CART) in the hypothalamus [38], [39]. However, in the hypermetabolic ELOVL3-ablated mouse model, hypothalamic expression of POMC and CART were similar to the wild type despite the lean phenotype and lower food intake [14]. Anorexigenic neurons that express POMC and CART project to central melanocortin receptor (MCR)-expressing neurons. Disruption of this system leads to hyperphagia and reduced metabolic rates in mice [40], [41]. Additionally, our group has previously shown that humans with a frameshift or missense mutation in the MC4R gene have lower metabolic rates [42]. Thus in light of the ELOVL3-ablated mouse model, it is possible that very-long-chain SAFAs regulate metabolic rate via inhibition of hypothalamic POMC expression and MC4R signaling. Region-specific ablation of ELOVL3 in the CNS in rodents may help elucidate this pathway.

Long-chain MUFAs (palmitoleic and oleic acid) in CSF were moderately associated (notwithstanding correction for multiple comparison) with lower 24 h RQ (indicating higher rates of lipid or protein oxidation) (**Figure 2**). RQ was measured in 15 minute intervals but no clear pattern of nocturnal or daytime RQ measurements were seen when results were examined by comparing the upper and lower strata (as defined by the median measurement) of oleic or palmitoleic acid (data not shown). Although these data did not survive strict Bonferroni correction, previous human clinical data support this observation. Kien et al. have shown that a diet intervention with diets high in oleic acid reduces the RQ [5], [43]. In this respect, Cha et al. have demonstrated that attenuation of central FA synthesis leads to a rapid increase in skeletal muscle lipid oxidation rates [20]. However, whether diet intervention effects on peripheral substrate oxidation involve central fatty acid metabolism remains speculative and still awaits exploration.

**Figure 4:**
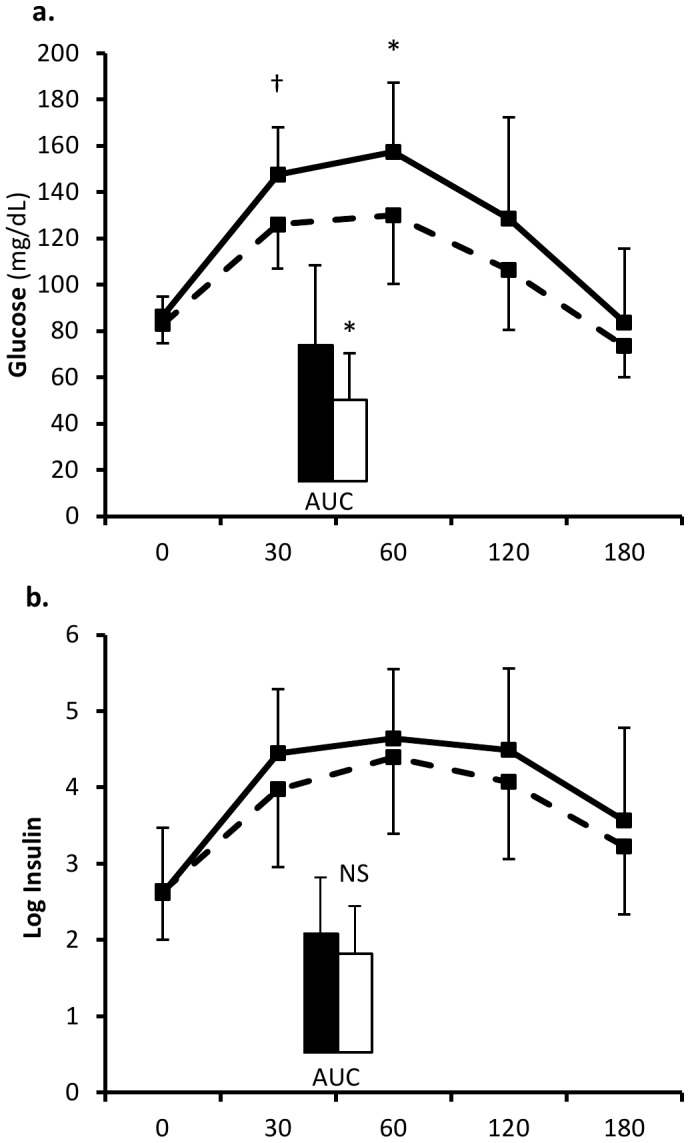
Glucose and insulin levels during the OGTT. Glucose (a.) and insulin (b.) levels are shown over the time course of the OGTT. Dashed lines and open bars represent individuals with high palmitoleic acid (PA) in the CSF, full lines and closed bars represent low PA in the CSF. Groups were defined as PA>0.63 (high) and PA<0.63 (low), where 0.63 is the median of central PA representation. Error bars depict standard deviation. AUC: area under the curve; *p<0.05; †p<0.01.

Finally, palmitoleic and oleic acid were also associated with lower glucose area under the curve during the OGTT (**Figure 2** **,** **3c**). These data support the initial hypothesis that central long-chain MUFAs are linked with peripheral insulin-mediated glucose utilization and are thus associated with lower blood glucose concentrations. Stratifying the study group into a high and low palmitoleic acid (PA) group, **Figure 4** **A** shows that the high PA group had lower glucose levels during the OGTT, however insulin levels were not significantly different between the groups (**Figure 4** **B**). Therefore, we calculated the insulin sensitivity index (ISI, see [44]) which correlated positively with the representation of central palmitoleic acid (P = 0.02) and oleic acid (P = 0.03), even after adjustments for age, sex and body fat percentage. These data indicate that individuals with higher central palmitoleic and oleic acid may be more insulin sensitive. Indeed, in rodent experiments the effect of central oleic acid infusion on glucose utilization in peripheral organs has been demonstrated repeatedly together with associated increases in hypothalamic expression of anorectic neuropeptide expression [16], [18], [19]. In light of our data, these rodent experiments where single FAs are centrally injected are crucial to further the knowledge on the potential role of FA species as regulatory messengers in the CNS. However, in these previous rodent experiments it was possible to inject supraphysiologic concentrations of individual FAs and measure a specific metabolic response. We believe our data confirm these studies and expand on them as we demonstrate associations of FAs in the physiologic range with metabolic traits. As with the rodent studies, our results still indicate that specific FAs may serve as signals to control key aspects of metabolism, but expand on the pool of FAs that may have a role in regulating each trait. Future human intervention studies need to test causality of such associations.

Obici et al. have suggested a K^+^-channel-mediated mechanism in the CNS for this pathway [18]. To our knowledge, in humans no data are available on central FAs and glucose concentrations during an OGTT. However, recent studies have shown positive associations between circulating palmitoleic acid and insulin sensitivity and lower incidence of diabetes [2], [45]. Chronic administration of palmitoleic acid in mice also reduced insulin resistance and hepatic lipid accumulation [46]. Various PUFAs also correlated with lower GAUC such as arachidonic acid (20∶4ω6), the product of the delta-5-desaturase. Although not shown in neuronal tissues, Borkman et al. have demonstrated associations between insulin sensitivity and muscle phospholipid-derived arachidonic acid. These data indirectly support our observation of a negative association of central abundance of arachidonic acid with GAUC [47]. In mice, Lam et al. have demonstrated that under hyperlipidemic conditions hypothalamic sensing of FAs is required for peripheral glucose homeostasis. This occurs via coenzyme-A-esterified FAs which activate hypothalamic K^+^-channels and recruit vagal efferents to the liver controlling hepatic glucose production [15]. Together, based on our results and existing literature, it may be possible that besides central oleic and palmitoleic acid, central long-chain FAs with a higher degree of desaturation may also be important candidates for central regulation of peripheral glucose utilization in humans. Nevertheless, we cannot exclude the possibility that a change in metabolism may itself lead to altered FA representation in the CNS.

One limiting factor of our study is the relatively low number of study subjects. However, CSF is difficult to obtain thus limiting sample size of such studies. Furthermore, we measured FA profiles in CSF and not brain tissue. Thus, conclusions are based on the assumption that FA in CSF are linked to peripheral metabolic traits and we therefore can only speculate on brain tissue concentrations and signal transduction pathways in the brain tissue itself. It also must be acknowledged that due to the large number of measurements, lipidomic studies are subject to false discovery error due to multiple comparisons. Nevertheless, some of the associations in this analysis were robust to the conservative Bonferonni correction for multiple comparisons and at the same time consistent with data previously presented in the literature. Additionally, the presented data are limited by potential covariation of metabolic traits with menstrual cycle state in women which was not accounted for. It also has to be acknowledged that the presented data are of cross-sectional nature and thus represent correlations that do not allow for proof of causality.

Altogether, FA species in the CNS are associated with metabolic traits such as energy expenditure, plasma glucose and substrate utilization in humans which is consistent with previous research in rodents. These data set the ground for future intervention experiments to test whether central FAs have the potential to regulate peripheral metabolism in humans or whether the opposite is the case.

## References

[1] Das UN (2006). Essential fatty acids: biochemistry, physiology and pathology.. Biotechnol J.

[2] Stefan N, Kantartzis K, Celebi N, Staiger H, Machann J (2010). Circulating palmitoleate strongly and independently predicts insulin sensitivity in humans.. Diabetes Care.

[3] Warensjo E, Riserus U, Vessby B (2005). Fatty acid composition of serum lipids predicts the development of the metabolic syndrome in men.. Diabetologia.

[4] Warensjo E, Ohrvall M, Vessby B (2006). Fatty acid composition and estimated desaturase activities are associated with obesity and lifestyle variables in men and women.. Nutr Metab Cardiovasc Dis.

[5] Kien CL, Bunn JY, Ugrasbul F (2005). Increasing dietary palmitic acid decreases fat oxidation and daily energy expenditure.. Am J Clin Nutr.

[6] Ohrvall M, Sundlof G, Vessby B (1996). Gamma, but not alpha, tocopherol levels in serum are reduced in coronary heart disease patients.. J Intern Med.

[7] Pan DA, Lillioja S, Milner MR, Kriketos AD, Baur LA (1995). Skeletal muscle membrane lipid composition is related to adiposity and insulin action.. J Clin Invest.

[8] Kawashima A, Sugawara S, Okita M, Akahane T, Fukui K (2009). Plasma fatty acid composition, estimated desaturase activities, and intakes of energy and nutrient in Japanese men with abdominal obesity or metabolic syndrome.. J Nutr Sci Vitaminol (Tokyo).

[9] van den Berg SA, Guigas B, Bijland S, Ouwens M, Voshol PJ (2010). High levels of dietary stearate promote adiposity and deteriorate hepatic insulin sensitivity.. Nutr Metab (Lond).

[10] Warensjo E, Riserus U, Gustafsson IB, Mohsen R, Cederholm T (2008). Effects of saturated and unsaturated fatty acids on estimated desaturase activities during a controlled dietary intervention.. Nutr Metab Cardiovasc Dis.

[11] Zhou YE, Egeland GM, Meltzer SJ, Kubow S (2009). The association of desaturase 9 and plasma fatty acid composition with insulin resistance-associated factors in female adolescents.. Metabolism.

[12] Jakobsson A, Westerberg R, Jacobsson A (2006). Fatty acid elongases in mammals: their regulation and roles in metabolism.. Prog Lipid Res.

[13] Kitazawa H, Miyamoto Y, Shimamura K, Nagumo A, Tokita S (2009). Development of a high-density assay for long-chain fatty acyl-CoA elongases.. Lipids.

[14] Zadravec D, Brolinson A, Fisher RM, Carneheim C, Csikasz RI (2010). Ablation of the very-long-chain fatty acid elongase ELOVL3 in mice leads to constrained lipid storage and resistance to diet-induced obesity.. FASEB J.

[15] Lam TK, Pocai A, Gutierrez-Juarez R, Obici S, Bryan J (2005). Hypothalamic sensing of circulating fatty acids is required for glucose homeostasis.. Nat Med.

[16] Ross RA, Rossetti L, Lam TK, Schwartz GJ (2010). Differential effects of hypothalamic long-chain fatty acid infusions on suppression of hepatic glucose production.. Am J Physiol Endocrinol Metab.

[17] Haywood SC, Bree AJ, Puente EC, Daphna-Iken D, Fisher SJ (2009). Central but not systemic lipid infusion augments the counterregulatory response to hypoglycemia.. Am J Physiol Endocrinol Metab.

[18] Obici S, Feng Z, Morgan K, Stein D, Karkanias G (2002). Central administration of oleic acid inhibits glucose production and food intake.. Diabetes.

[19] Schwinkendorf DR, Tsatsos NG, Gosnell BA, Mashek DG (2011). Effects of central administration of distinct fatty acids on hypothalamic neuropeptide expression and energy metabolism.. Int J Obes (Lond).

[20] Cha SH, Hu Z, Chohnan S, Lane MD (2005). Inhibition of hypothalamic fatty acid synthase triggers rapid activation of fatty acid oxidation in skeletal muscle.. Proc Natl Acad Sci U S A.

[21] Cha SH, Rodgers JT, Puigserver P, Chohnan S, Lane MD (2006). Hypothalamic malonyl-CoA triggers mitochondrial biogenesis and oxidative gene expression in skeletal muscle: Role of PGC-1alpha.. Proc Natl Acad Sci U S A.

[22] Kumar MV, Shimokawa T, Nagy TR, Lane MD (2002). Differential effects of a centrally acting fatty acid synthase inhibitor in lean and obese mice.. Proc Natl Acad Sci U S A.

[23] Thupari JN, Landree LE, Ronnett GV, Kuhajda FP (2002). C75 increases peripheral energy utilization and fatty acid oxidation in diet-induced obesity.. Proc Natl Acad Sci U S A.

[24] Ravussin E, Lillioja S, Anderson TE, Christin L, Bogardus C (1986). Determinants of 24-hour energy expenditure in man. Methods and results using a respiratory chamber.. J Clin Invest.

[25] Cadas H, di TE, Piomelli D (1997). Occurrence and biosynthesis of endogenous cannabinoid precursor, N-arachidonoyl phosphatidylethanolamine, in rat brain.. J Neurosci.

[26] Fu J, Astarita G, Gaetani S, Kim J, Cravatt BF (2007). Food intake regulates oleoylethanolamide formation and degradation in the proximal small intestine.. J Biol Chem.

[27] Giuffrida A, Rodriguez de FF, Piomelli D (2000). Quantification of bioactive acylethanolamides in rat plasma by electrospray mass spectrometry.. Anal Biochem.

[28] Schwartz GJ, Fu J, Astarita G, Li X, Gaetani S (2008). The lipid messenger OEA links dietary fat intake to satiety.. Cell Metab.

[29] Weyer C, Snitker S, Rising R, Bogardus C, Ravussin E (1999). Determinants of energy expenditure and fuel utilization in man: effects of body composition, age, sex, ethnicity and glucose tolerance in 916 subjects.. Int J Obes Relat Metab Disord.

[30] Jumpertz R, Guijarro A, Pratley RE, Piomelli D, Krakoff J (2011). Central and peripheral endocannabinoids and cognate acylethanolamides in humans: association with race, adiposity, and energy expenditure.. J Clin Endocrinol Metab.

[31] Smedman AE, Gustafsson IB, Berglund LG, Vessby BO (1999). Pentadecanoic acid in serum as a marker for intake of milk fat: relations between intake of milk fat and metabolic risk factors.. Am J Clin Nutr.

[32] Cunnane SC, Schneider JA, Tanqney C, Tremblay-Mercier J, Fortier M (2012). Plasma and brain fatty acid profiles in mild cognitive impairment and Alzheimer’s disease.. J Alzheimers Dis.

[33] Mitchell RW, Hatch GM (2011). Fatty acid transport into the brain: of fatty acid fables and lipid tails.. Prostaglandins Leukot Essent Fatty Acids.

[34] Edmond J (2001). Essential polyunsaturated fatty acids and the barrier to the brain: the components of a model for transport.. J Mol Neurosci.

[35] Dhopeshwarkar GA, Subramanian C, McConnell DH, Mead JF (1972). Fatty acid transport into the brain.. Biochim Biophys Acta.

[36] Esposito G, Giovacchini G, Liow JS, Bhattacharjee AK, Greenstein D (2008). Imaging neuroinflammation in Alzheimer’s disease with radiolabeled arachidonic acid and PET.. J Nucl Med.

[37] Chen CT, Green JT, Orr SK, Bazinet RP (2008). Regulation of brain polyunsaturated fatty acid uptake and turnover.. Prostaglandins Leukot Essent Fatty Acids.

[38] Kristensen P, Judge ME, Thim L, Ribel U, Christjansen KN (1998). Hypothalamic CART is a new anorectic peptide regulated by leptin.. Nature.

[39] Mizuno TM, Makimura H, Silverstein J, Roberts JL, Lopingco T (1999). Fasting regulates hypothalamic neuropeptide Y, agouti-related peptide, and proopiomelanocortin in diabetic mice independent of changes in leptin or insulin.. Endocrinology.

[40] Huszar D, Lynch CA, Fairchild-Huntress V, Dunmore JH, Fang Q (1997). Targeted disruption of the melanocortin-4 receptor results in obesity in mice.. Cell.

[41] Ste ML, Miura GI, Marsh DJ, Yagaloff K, Palmiter RD (2000). A metabolic defect promotes obesity in mice lacking melanocortin-4 receptors.. Proc Natl Acad Sci U S A.

[42] Krakoff J, Ma L, Kobes S, Knowler WC, Hanson RL (2008). Lower metabolic rate in individuals heterozygous for either a frameshift or a functional missense MC4R variant.. Diabetes.

[43] Kien CL, Bunn JY (2007). Effects of palmitate and oleate on the respiratory quotient during acute feeding.. Obesity (Silver Spring).

[44] Cederholm J, Wibell L (1990). Insulin release and peripheral sensitivity at the oral glucose tolerance test.. Diabetes Res Clin Pract.

[45] Mozaffarian D, Cao H, King IB, Lemaitre RN, Song X (2010). Trans-palmitoleic acid, metabolic risk factors, and new-onset diabetes in U.S. adults: a cohort study.. Ann Intern Med.

[46] Yang ZH, Miyahara H, Hatanaka A (2011). Chronic administration of palmitoleic acid reduces insulin resistance and hepatic lipid accumulation in KK-Ay Mice with genetic type 2 diabetes.. Lipids Health Dis.

[47] Borkman M, Storlien LH, Pan DA, Jenkins AB, Chisholm DJ (1993). The relation between insulin sensitivity and the fatty-acid composition of skeletal-muscle phospholipids.. N Engl J Med.

